# Poor adherence to guideline-directed anticoagulation in elderly Chinese patients with atrial fibrillation: a report from the Optimal Thromboprophylaxis in Elderly Chinese Patients with Atrial Fibrillation (ChiOTEAF) registry

**DOI:** 10.1093/ehjqcco/qcab054

**Published:** 2021-08-09

**Authors:** Yutao Guo, Agnieszka Kotalczyk, Jacopo F Imberti, Yutang Wang, Gregory Y H Lip, ChiOTEAF Registry Investigators

**Affiliations:** Department of Pulmonary Vessel and Thrombotic Disease, Sixth Medical Centre, Chinese PLA General Hospital, Beijing 100142, China; Liverpool Centre for Cardiovascular Science, University of Liverpool and Liverpool Heart and Chest Hospital, Liverpool, UK; Liverpool Centre for Cardiovascular Science, University of Liverpool and Liverpool Heart and Chest Hospital, Liverpool, UK; Department of Cardiology, Congenital Heart Diseases and Electrotherapy, Medical University of Silesia, Silesian Centre for Heart Diseases, Zabrze, Poland; Liverpool Centre for Cardiovascular Science, University of Liverpool and Liverpool Heart and Chest Hospital, Liverpool, UK; Cardiology Division, Department of Biomedical, Metabolic and Neural Sciences, University of Modena and Reggio Emilia, Policlinico di Modena, Modena, Italy; Department of Cardiology, Second Medical Centre, Chinese PLA General Hospital, Beijing 100853, China; Department of Pulmonary Vessel and Thrombotic Disease, Sixth Medical Centre, Chinese PLA General Hospital, Beijing 100142, China; Liverpool Centre for Cardiovascular Science, University of Liverpool and Liverpool Heart and Chest Hospital, Liverpool, UK; Aalborg Thrombosis Research Unit, Department of Clinical Medicine, Aalborg University, Aalborg, Denmark; Department of Pulmonary Vessel and Thrombotic Disease, Sixth Medical Centre, Chinese PLA General Hospital, Beijing 100142, China

**Keywords:** Atrial fibrillation, Oral anticoagulation, Vitamin K antagonists, Non-vitamin K oral anticoagulants, Registry, Prognosis

## Abstract

**Aims:**

Adherence to guideline-directed oral anticoagulation (OAC) in patients with atrial fibrillation (AF) improves outcomes, but limited data are available from China. We evaluated the adherence to guideline-directed anticoagulation and its impact on clinical outcomes in a high-risk cohort of elderly Chinese patients.

**Methods and results:**

The Optimal Thromboprophylaxis in Elderly Chinese Patients with Atrial Fibrillation (ChiOTEAF) registry is a prospective, multicentre study conducted from October 2014 to December 2018. Endpoints of interest were all-cause death, thromboembolic (TE) events and major bleedings in patients with a guideline-directed indication for OACs (CHA_2_DS_2_-VASc ≥1 if male or ≥2 if female). The eligible cohort consisted of 5742 patients, of whom 2567 (44.7%) patients were treated with an OAC. Seven independent predictors of OAC undertreatment were identified: age [odds ratio (OR): 1.04; 95% confidence interval (CI): 1.03–1.05; *P* < 0.001], first diagnosed AF (OR: 1.71; 95%CI: 1.44–2.03; *P* < 0.001), chronic kidney disease (OR: 1.67; 95%CI: 1.36–2.06; *P* < 0.001), liver disease (OR: 1.69; 95%CI: 1.19–2.41; *P* = 0.003), dementia (OR: 1.67; 95%CI: 1.06–2.64; *P* = 0.026), prior extracranial bleeding (OR: 1.89; 95%CI: 1.35–2.64; *P* < 0.001), and the use of antiplatelet drug (OR: 6.97; 95%CI: 5.89–8.23; *P* < 0.001). On multivariate analysis, OAC undertreatment was significantly associated with a higher risk all-cause death (OR: 3.79; 95%CI: 2.61–5.53; *P* < 0.001) and TE events (OR: 2.28; 95%CI: 1.39–3.72; *P* = 0.001), and a similar risk of major bleeding as compared with guideline-directed OAC therapy.

**Conclusion:**

Only 44.7% of all eligible patients were prescribed OAC in accordance with guideline recommendations. The independent predictors for OAC undertreatment were age, first diagnosed AF, chronic kidney disease, chronic obstructive pulmonary disease, prior extracranial bleeding, and the use of the antiplatelet drugs. Guideline-adherent thromboprophylaxis was safe and may be associated with improved survival and less TE among elderly Chinese patients with AF.

## Graphical Abstract

**Graphical Abstract:**
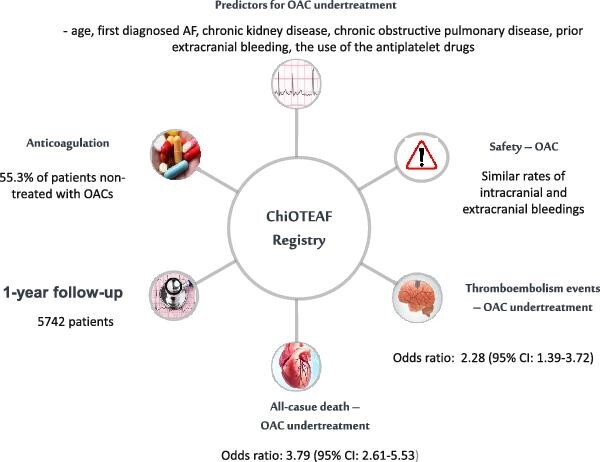
Poor adherence to guideline-directed anticoagulation in elderly Chinese patients with atrial fibrillation. OAC oral anticoagulant; CI - confidence interval

## Introduction

Stroke prevention is central to the management of patients with atrial fibrillation (AF), even as part of a holistic or integrated approach to AF patient care^1^—as currently promoted in guidelines.^2^ Indeed, oral anticoagulation (OAC) therapy is recommended for stroke prevention in AF patients, except for those with a very low risk of ischaemic stroke.^2,3^

However, previous studies have reported poor adherence to practice guidelines, especially evident in Asian patients with AF.^4,5^ At the same time, patients who received guideline-adherent treatment had better outcomes compared with those not treated in accordance with the guideline recommendations.^6–10^ On the contrary, there is an inherent fear of OAC-related bleeding which limits OAC uptake in Chinese patients,^11^ yet many of these patients are prescribed with antiplatelet agents.^12,13^ Whilst warfarin was associated with a high risk of major bleeding and intracranial haemorrhage in Asians, compared with non-Asians, the non-vitamin K antagonist oral anticoagulants (NOACs) may offer better safety, efficacy, and convenience in Asians.^14^ Nevertheless, data on the importance of guideline-directed thromboprophylaxis in the era of NOACs for Chinese AF patients are still limited.

The prospective, nationwide Optimal Thromboprophylaxis in Elderly Chinese Patients with Atrial Fibrillation (ChiOTEAF) registry aimed to explore contemporary regional AF management strategies, focusing on antithrombotic therapy. In this analysis, we first evaluated the adherence to guideline-directed anticoagulation and second the impact of guideline adherence on clinical outcomes in a high-risk cohort of elderly Chinese patients with AF.

## Methods

### Study design

The ChiOTEAF registry is a prospective, observational, large-scale multicentre registry conducted between October 2014 and December 2018 in 44 sites from 20 provinces in China. The study protocol has been previously published.^15^ In brief, consecutive patients presenting to cardiologists, neurologists, or surgeons with a documented AF episode within 12 months were enrolled. Follow-up was performed at 6 and 12 months and then annually for the next two years. Data were collected at enrolment and follow-up visits by local investigators. The registry was approved by the Central Medical Ethics Committee of Chinese PLA General Hospital, Beijing, China (approval no S2014-065-01) and local institutional review boards.

### Definitions and study cohort

Variables included in the registry and their definitions were designed to match the EURObservational Research Programme Atrial Fibrillation (EORP-AF) Long-term General Registry.^16^ The CHA_2_DS_2_-VASc score^17^ [congestive heart failure or left ventricular dysfunction, hypertension, age ≥75 years (doubled), diabetes, stroke (doubled), vascular disease, age 65–74 years, female sex] and the HAS-BLED bleeding score^18^ [hypertension, abnormal renal/liver function, stroke, bleeding history or predisposition, the labile international normalized ratio (INR), elderly, drugs/alcohol use] were used to assess the thromboembolic (TE) and bleeding risks. Bleeding events were categorized according to the International Society on Thrombosis and Haemostasis (ISTH) definition.^19^ Stroke and transient ischaemic attack were defined according to the World Health Organization definition^20^ and American Heart Association/American Stroke Association Stroke Council statement.^21^ A guideline-directed indication for OACs was defined based on the 2020 ESC guidelines as CHA_2_DS_2_-VASc ≥1 if male or ≥2 if female and guideline-directed thromboprophylaxis as using a vitamin K antagonist (VKA) or NOAC among those patients.^2^ Not-using OACs among patients with a guideline-directed indication was defined as ‘undertreatment’ (non-OAC group).

All patients included in the analysis were aged ≥65 years, had a guideline-adherent indication for antithrombotic therapy, and available data on the use of OAC and follow-up.

### Study outcomes

The objectives of the analysis were (i) to describe the patterns and persistence of anticoagulation in elderly Chinese patients; (ii) to identify potential predictors of OAC non-use; and (iii) to evaluate the impact of guideline-directed anticoagulation on clinical outcomes, including TE events (ischaemic stroke, transient ischaemic attack, or peripheral embolism), major bleedings (intracranial and extracranial bleedings), and all-cause death.

### Statistical analysis

Continuous variables are expressed as mean ± standard deviation (SD) and categorical variables as counts and percentages. Between-group comparisons were made by using a chi-square test or a Fisher's exact test if any expected cell count was less than five. A logistic univariate regression analysis was used to assess the predictors of OAC non-use. Characteristics significantly (*P* < 0.05) associated with OAC non-use were subsequently entered in a multivariate regression model. Finally, the association between OAC undertreatment and outcomes (TE events, major bleeding, and death) was assessed by logistic univariate regression analysis. We provided additional analysis for OAC non-use and outcomes only for patients with CHA_2_DS_2_-VASc ≥2 if male and ≥3 if female.

In all analyses, a *P-*value < 0.05 was considered statistically significant. Statistical analysis was performed using SPSS® version 24 (IBM Corp., Armonk, NY, USA).

## Results

The ChiOTEAF registry enrolled 7077 patients, of whom 657 (9.3%) were lost to follow-up. The eligible cohort for this analysis included 5742 patients, of whom the majority were male (59.4%), with mean age of 75.4 ± 10 years and a high risk of stroke (mean CHA_2_DS_2_-VASc score: 3.7 ± 1.6). We identified 3175 (55.3%) patients, who were not taking OACs (non-OAC group) and 2567 (44.7%) patients treated with a VKA or NOAC (OAC group). Patients included in the non-OAC group were older (77.2 vs. 73.1 years; *P* < 0.001) and with a higher incidence of comorbidities, in particular diabetes (29.2% vs. 26.0%; *P* = 0.008), coronary artery disease (56.0% vs. 41.2%; *P* < 0.001), chronic kidney disease (8.2% vs. 4.0%; *P* < 0.001), liver disease (4.3% vs. 3.0%), chronic obstructive pulmonary disease (11.2% vs. 6.6%; *P* < 0.001), and dementia (3.8% vs. 2.1%; *P* < 0.001) compared with the anticoagulated patients. The baseline characteristics are reported in *Table 1*.

**Table 1:** Baseline characteristics

	Total	Non-OAC**^a^**	OAC^b^	
	(*N* = 5742)	(*N* = 3175)	(*N* = 2567)	
	*n* (%)	*n* (%)	*n* (%)	*P*-value
Age,^c^ years	75.4 ± 10	77.2 ± 9.7	73.1 ± 10	<0.001
Female gender	2331 (40.6)	1224 (38.6)	1107 (43.1)	<0.001
First diagnosed AF (*n* = 4902)	848 (17.3)	549 (20.9)	299 (13.2)	<0.001
Diabetes (*n* = 5741)	1592 (27.7)	925 (29.2)	667 (26.0)	0.008
Hypertension (*n* = 5741)	3846 (67.0)	2142 (67.5)	1704 (66.4)	0.397
Heart failure (*n* = 5741)	2102 (36.6)	1189 (37.4)	913 (35.6)	0.144
Coronary artery disease (*n* = 5628)	2779 (49.4)	1740 (56.0)	1039 (41.2)	<0.001
Ischaemic stroke	517 (9.0)	287 (9.0)	230 (9.0)	0.917
Chronic kidney disease (*n* = 5721)	698 (12.2)	469 (8.2)	229 (4.0)	<0.001
Liver disease (*n* = 5715)	211 (3.7)	135 (4.3)	76 (3.0)	0.010
COPD	525 (9.1)	356 (11.2)	169 (6.6)	<0.001
Sleep apnoea (*n* = 5621)	198 (3.5)	104 (3.4)	94 (3.7)	0.462
Dementia	176 (3.1)	121 (3.8)	55 (2.1)	<0.001
CHA_2_DS_2_-VASc^c^	3.7 ± 1.6	3.8 ± 1.7	3.7 ± 1.6	0.003
CHA_2_DS_2_-VASc = 1 if male or ≤ 2 if female	618 (10.8)	324 (10.2)	294 (11.5)	0.129
CHA_2_DS_2_-VASc ≥ 2 if male or ≥ 3 if female	5124 (89.2)	2851 (89.8)	2273 (88.5)	0.129
HAS-BLED^c^	2.2 ± 1.1	2.4 ± 1.1	2.0 ± 1.0	<0.001
Intracranial bleeding (*n* = 5721)	125 (2.2)	82 (2.6)	43 (1.7)	0.019
Extracranial bleeding (*n* = 5723)	230 (4.0)	147 (4.6)	83 (3.2)	0.007
Antiplatelet (*n* = 5730)	2433 (42.4)	2055 (64.8)	378 (14.8)	<0.001
Left atrial appendage occlusion (*n* = 5741)	17 (0.3)	7 (0.2)	10 (0.4)	0.241

AF, atrial fibrillation; CHA_2_DS_2_-VASc, congestive heart failure or left ventricular dysfunction, hypertension, age ≥ 75 years (doubled), diabetes, stroke (doubled), vascular disease, age 65–74 years, female sex; COPD, chronic obstructive pulmonary disease; HAS-BLED, hypertension, abnormal renal/liver function, stroke, bleeding history or predisposition, labile international normalized ratio, elderly, drugs/alcohol use.

a Oral anticoagulant undertreatment

^b^Guideline-directed OAC therapy

^c^Mean ± standard deviation

### Anticoagulation patterns and persistence

**Figure 1:**
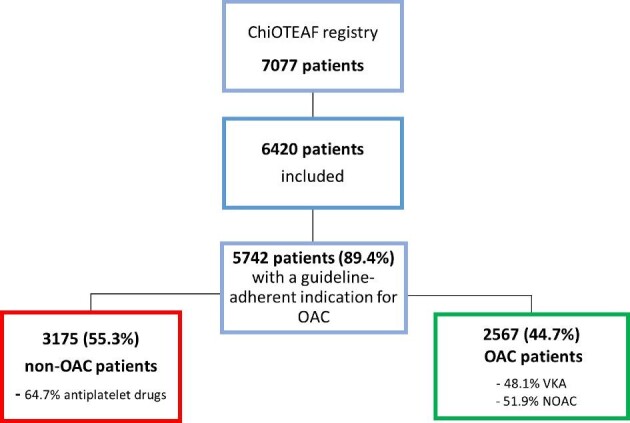
Flowchart of patient inclusion. ChiOTEAF, Optimal Thromboprophylaxis in Elderly Chinese Patients with Atrial Fibrillation registry; NOAC, non-vitamin K antagonist, OAC, oral anticoagulation; VKA, vitamin K antagonist.

Among patients included in the OAC group, the proportions of those treated with NOACs and VKAs were similar (51.9% and 48.1%, respectively). The majority of NOAC-treated patients received dabigatran (70.9%) or rivaroxaban (27.7%). Among patients included in the non-OAC group, 2055 (64.7%) were treated with antiplatelet agents (*Figure 1*).

**Figure 2:**
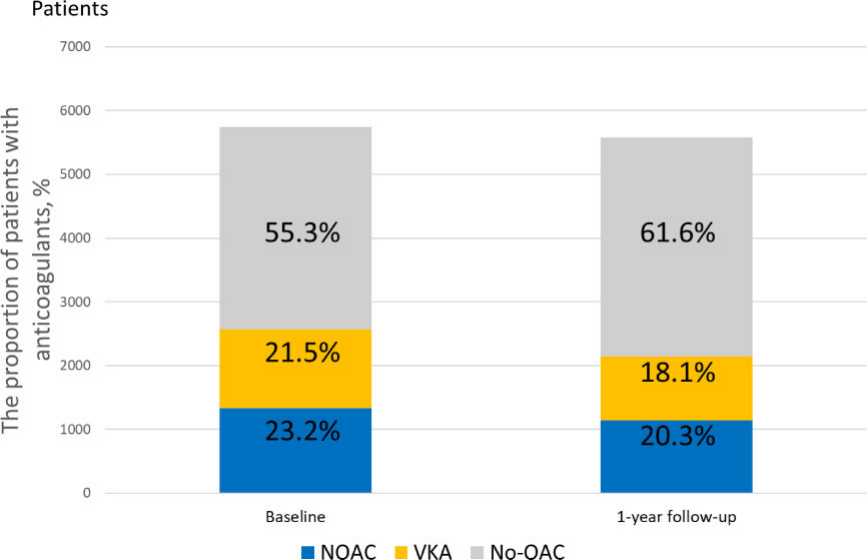
Proportions of patients with a guideline-adherent anticoagulation. NOAC, non-vitamin K antagonist; OAC, oral anticoagulation; VKA, vitamin K antagonist.

**Figure 3:**
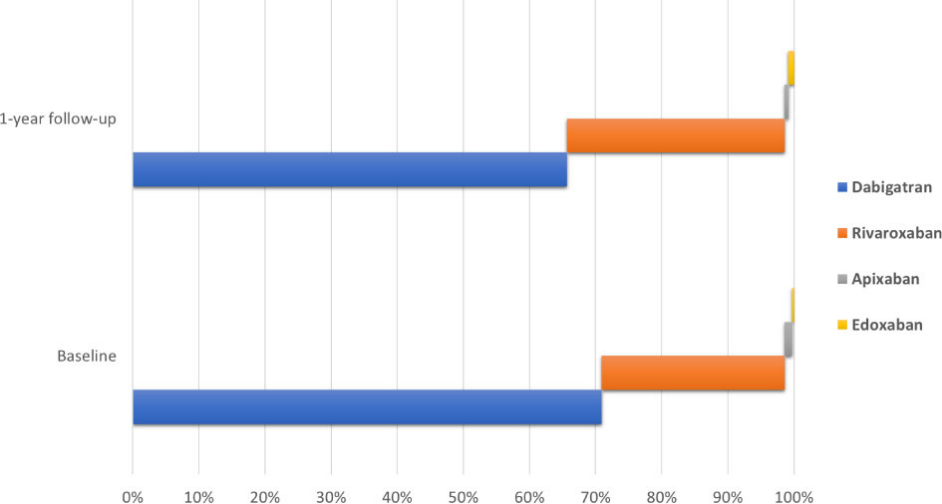
Proportions of patients treated with dabigatran, rivaroxaban, apixaban, and edoxaban.

Data on the persistence of OAC therapy during a 1-year follow-up were available for 5579 (97.2%) patients. Only 2144 (38.4%) patients received OACs, with a higher proportion of those treated with a NOAC (47.1% VKA vs. 52.9% NOAC; *Figure 2*). When compared with baseline, more patients were treated with rivaroxaban (32.9% vs. 27.7%), while dabigatran was used in 65.7% of the NOAC-treated patients at 1-year follow-up (*Figure 3*).

### Multivariate analysis

On multivariate analysis (*Table 2*), seven independent predictors of OAC undertreatment were identified: age (OR: 1.04; 95%CI: 1.03–1.05; *P* < 0.001), first diagnosed AF (OR: 1.71; 95%CI: 1.44–2.03; *P* < 0.001), chronic kidney disease (OR: 1.67; 95%CI: 1.36–2.06; *P* < 0.001), liver disease (OR: 1.69; 95%CI: 1.19–2.41; *P* = 0.003), dementia (OR: 1.67; 95%CI: 1.06–2.64; *P* = 0.026), prior extracranial bleeding (OR: 1.89; 95%CI: 1.35–2.64; *P* < 0.001), and the use of an antiplatelet drug (OR: 6.97; 95%CI: 5.89–8.23; *P* < 0.001). On contrary, women were more likely to receive OACs (OR: 0.82; 95%CI: 0.72–0.93; *P* < 0.001).

**Table 2:** Predictors of oral anticoagulant undertreatment

	Univariate			Multivariate		
	Odds ratio	95%CI	*P*-value	Odds ratio	95%CI	*P*-value
Age	1.04	1.04–1.05	<0.001	1.04	1.03–1.05	<0.001
Female gender	0.83	0.74–0.92	<0.001	0.82	0.72–0.93	<0.001
First diagnosed AF	1.74	1.49–2.02	<0.001	1.71	1.44–2.03	<0.001
Diabetes	1.17	1.04–1.32	0.008	—	—	—
Hypertension	1.05	0.94–1.17	0.397			
Heart failure	1.08	0.97–1.21	0.144			
Coronary artery disease	1.82	1.64–2.03	<0.001	—	—	—
Ischaemic stroke	1.01	0.84–1.21	0.917			
Chronic kidney disease	1.77	1.50–2.09	<0.001	1.67	1.36–2.06	<0.001
Liver disease	1.46	1.09–1.94	0.010	1.69	1.19–2.41	0.003
COPD	1.79	1.48–2.17	<0.001	—	—	—
Sleep apnoea	0.89	0.68–1.19	0.462			
Dementia	1.81	1.31–2.50	<0.001	1.67	1.06–2.64	0.026
Intracranial bleeding	1.56	1.07–2.26	0.020	—	—	—
Extracranial bleeding	1.45	1.10–1.91	0.008	1.89	1.35–2.64	<0.001
Antiplatelet	10.65	9.34–12.15	<0.001	6.97	5.89–8.23	<0.001

AF, atrial fibrillation; CI, confidence interval; COPD, chronic obstructive pulmonary disease.

### Clinical outcomes

During 1-year follow-up, 83 TE events (1.5%) and 188 deaths (3.3%) occurred. OAC undertreatment was significantly associated with all-cause death (OR: 3.79; 95%CI: 2.61–5.53; *P* < 0.001) and TE events (OR: 2.28; 95%CI: 1.39–3.72; *P* = 0.001) (*Table 3*). In terms of OAC safety, 69 major bleedings were reported (10 intracranial bleedings and 59 extracranial bleedings), but no statistically significant differences were noted between groups (*Table 3*).

**Table 3:** Risk of adverse outcomes in the oral anticoagulant undertreatment (Non-OAC) group as compared to the guideline-directed therapy (OAC) group

	Non-OAC	OAC	
	(*N* = 3175)	(*N* = 2567)	Odds ratio
Outcome	*n* (%)	*n* (%)	(95%CI)
All-cause death	154 (4.9)	34 (1.3)	3.79 (2.61–5.53)
TE events	61 (1.9)	22 (0.9)	2.28 (1.39–3.72)
Intracranial bleeding	6 (0.2)	4 (0.2)	1.22 (0.34–4.32)
Extracranial major bleeding	36 (1.1)	23 (0.9)	1.27 (0.75–2.16)

CI, confidence interval; OAC, oral anticoagulation; TE, thromboembolic.

We performed additional analysis for patients with CHA_2_DS_2_-VASc score ≥2 if male and ≥3 if female. In this subgroup, OAC non-use was associated with a higher odds of all-cause death (OR: 3.90; 95%CI: 2.74–5.56) and TE events (OR: 2.54; 95%CI: 1.57–4.09) without a significant excess in major bleedings (*Table 4*).

**Table 4:** Risk of adverse outcomes in the untreated (Non-OAC) group as compared to the anticoagulated (OAC) group in patients with CHA_2_DS_2_-VASc score ≥2 if male and ≥3 if female

	Non-OAC	OAC	
	(*N* = 2851)	(*N* = 2891)	Odds ratio
Outcome	*n* (%)	*n* (%)	(95%CI)
All-cause death	148 (5.2)	40 (1.4)	3.90 (2.74–5.56)
TE events	59 (2.1)	24 (0.8)	2.54 (1.57–4.09)
Intracranial bleeding	6 (0.2)	4 (0.1)	1.53 (0.43–5.43)
Extracranial major bleeding	35 (1.2)	24 (0.8)	1.49 (0.89–2.51)

CI, confidence interval; OAC, oral anticoagulation; TE, thromboembolic.

## Discussion

The main findings of this analysis are as follows: (i) 55.3% of AF patients with a guideline-adherent indication for antithrombotic therapy were undertreated and 64.7% of these were treated with antiplatelet drugs; (ii) the independent predictors for OAC undertreatment were age, male sex, first diagnosed AF, chronic kidney disease, liver disease, dementia, prior extracranial bleeding, and the use of the antiplatelet drugs; and (iii) OAC undertreatment was significantly associated with a three-fold higher risk of all-cause death and two-fold higher risk of TE events, with a similar risk of major bleedings as compared with guideline-adherent OAC therapy.

The ChiOTEAF registry showed that 46.7% of patients were anticoagulated accordingly to the guidelines, with similar uptake of VKA (48.1%) and NOACs (51.9%) among Chinese elderly. Of note, all eligible patients had indications for OACs as one inclusion criterion was age ≥65 years. Even though the proportion of undertreated patients is higher than reported in the European registries,^9^ an improvement in the use of OACs among Chinese patients can be observed. The previously published Chinese Atrial Fibrillation Registry study showed that only 36.5% of AF patients with a CHA_2_DS_2_-VASc score ≥2 were anticoagulated.^22^ Furthermore, antiplatelet therapy was still used among 64.7% of the non-OAC patients, despite OACs having superior efficacy with similar major bleeding risks compared with aspirin among elderly patients with AF.^23,24^ The reasons of high antiplatelet use in Chinese patients are uncertain and non-evidence-based.

Of note, the Asian sub-analyses of four major randomized control trials of NOACs vs. VKA showed that standard-dose NOACs significantly reduced the risk of stroke/TE (HR: 0.65; 95%CI: 0.52–0.83) and major bleeding (HR: 0.57; 95%CI: 0.44–0.74), highlighting that the effect of reduction was even greater than in the non-Asian patients (HR: 0.85; 95%CI: 0.77–0.93 and HR: 0.89; 95%CI: 0.76–1.04, respectively).^25^

However, poor adherence of OACs (38.4%) at 1-year follow-up was reported in this study, with a slightly better persistence among patients treated with NOACs. To our knowledge, the ChiOTEAF registry is first to report the better persistence of the once-daily dosing regimen NOAC (rivaroxaban) compared with dabigatran (twice-daily regimen) in Chinese patients. Indeed, a recent meta-analysis showed that NOAC treatment was related to greater patient satisfaction as compared with VKAs^26^, and better persistence with NOACs compared with warfarin has also been shown in other registries.^27–30^ Consequently, a large cohort study of European AF patients reported lower persistence and adherence with dabigatran (persistence: 77%, adherence: 65%) compared with rivaroxaban (83% and 75%) during 1-year follow-up.^31^

We identified eight predictors of OAC undertreatment, including age, male sex, comorbidities (chronic kidney disease, liver disease, dementia, and prior extracranial bleeding), first AF episode, and antiplatelets use. Indeed, it may reflect the ‘real-world’ clinical practice, where the final choice of therapy is based on an individualized approach, considering several aspects of the clinical picture and not just the CHA_2_DS_2_-VASc score. However, age, multimorbidity, or first diagnosed AF should not be reasons for non-prescribing of OACs, but to pay attention to anticoagulation control (if on warfarin) or modify the dosage (e.g. for NOAC-treated patients with chronic kidney disease), according to the guidelines.^32,33^ The risk of ischaemic stroke in Asian patients with AF may be greater compared with non-Asians, even reaching the treatment threshold with OAC at age ≥55 years.^34,35^ Hence, early detection and implementation of therapy are crucial to avoid AF-related complications.^36^ Similarly, multi-morbidity was an independent factor of withholding OAC in the elderly.^37^ Nevertheless, a more holistic approach, including AF screening, appropriate evaluation and characterization of the arrhythmia, and treatment of comorbidities and cardiovascular risk factors, should be implemented.^38,39^ Indeed, a recent systematic review and meta-analysis found that compliance with the Atrial fibrillation Better Care pathway was associated with a lower risk of all-cause death (OR: 0.42; 95%CI: 0.31–0.56), cardiovascular death (OR: 0.37; 95%CI: 0.23–0.58), stroke (OR: 0.55; 95%CI: 0.37–0.82), and major bleeding (OR: 0.69; 95%CI: 0.51–0.94).^40^

In the present study, we found that OAC undertreatment was related to significantly poorer outcomes in the elderly, including a higher risk of all-cause death and TE. The risk of major bleeding was comparable between the two study groups, bearing in mind that over 60% of patients in the non-OAC group received antiplatelet agents. The guidelines recommend OAC for stroke prevention in AF patients with CHA_2_DS_2_-VASc ≥2 if men or ≥3 if women (class of recommendation I, Level of evidence A) and in AF patients with CHA_2_DS_2_-VASc ≥1 if male or ≥2 if female (class of recommendation IIa, level of evidence B), suggesting in the last setting a tailored treatment based on net clinical benefit and patients preferences.^2^ It is common practice prescribing OAC only for AF patients in case of CHA_2_DS_2_-VASc ≥2 if men or ≥3 if women. Of note, our study showed the advantage of OAC in both settings of recommendations. The survival advantage associated with OAC use and the absence of an excess bleeding risk emphasizes that guideline-adherent OAC therapy is safe and effective among Chinese elderly AF patients and that greater efforts should be made to increase OAC prescriptions.

### Limitations

The primary limitation of the study is its observational nature. Patients were enrolled in multiple centres with relatively long enrolment period, which may imply a potential variability in the local management for AF. The ChiOTEAF registry included patients between October 2014 and December 2018, and a guideline-directed indication for OACs were reviewed as defined based on the 2020 ESC guideline. The generalizability of the results may be limited as the study population was restricted to patients aged ≥65 years with available data on OAC therapy. The ‘real’ number of TE, major bleedings, and deaths may also be underestimated; 9.3% of patients were lost to follow-up, and the causes of 15.4% of deaths are unknown. These figures are comparable with other registries from Western countries.^41^ Given that only seven deaths and three TE events were reported in patients with CHA_2_DS_2_-VASc score 1 if male or 2 if female during the follow-up, we did not perform a separate analysis of this subgroup. Finally, neither data on anticoagulation control, inappropriate low doses of NOACs, and traditional Chinese medicines nor management of comorbidities were available and could not be considered in the analysis.

## Conclusions

Only 44.7% of all eligible patients were prescribed OAC in accordance with guideline recommendations. The independent predictors for OAC undertreatment were age, first diagnosed AF, chronic kidney disease, chronic obstructive pulmonary disease, prior extracranial bleeding, and the use of the antiplatelet drugs. Notwithstanding the observational nature of our study, guideline-adherent thromboprophylaxis appears to be safe and may be associated with improved survival and less TE among elderly Chinese patients with AF.

## Supplementary Material

qcab054_Supplemental_File

## Data availability

The datasets used and analysed during the current study are available from the corresponding author on reasonable request.
